# The Impact of Telemedicine Interventions on Maternal and Fetal Outcomes in Women With Gestational Diabetes: Systematic Review and Meta-Analysis of Randomized Controlled Trials

**DOI:** 10.2196/103569

**Published:** 2026-10-23

**Authors:** Hongyue He, Juan Deng, Jingxian Liu, Xiaohong Xu, Jiao Chen, Lixin Liu, Haoyue Zhang, Fang Wang

**Affiliations:** 1 School of Nursing, Chengdu University of Traditional Chinese Medicine, No. 37, Shierqiao RoadChengdu, Sichuan, China; 2 Department of Orthopedics, Hospital of Chengdu University of Traditional Chinese Medicine, No. 39, Shierqiao RoadChengdu, Sichaun, China; 3 School of Medicine, University of Electronic Science and Technology of China, No. 4, Section 2, Jianshe North RoadChengdu, Sichuan, China; 4 Dean's Office, Guang'an Hospital of Traditional Chinese Medicine, No. 1 Cuiping RoadGuang'an, Sichuan, 638099, China, 86 15520799476

**Keywords:** gestational diabetes mellitus, telemedicine, maternal and fetal outcomes, systematic review, meta-analysis

## Abstract

**Background:**

Gestational diabetes mellitus (GDM) has a rising global prevalence and brings short-term and long-term adverse risks for mothers and fetuses. Conventional in-person GDM care is limited by time and geographic barriers, often resulting in suboptimal compliance and delayed individualized interventions. Telemedicine is increasingly applied in GDM management; however, randomized controlled trial (RCT) evidence remains inconsistent.

**Objective:**

The study aims to pool data from RCTs to quantify the effects of telemedicine on glycemic control and pregnancy-related outcomes in women with GDM and to inform optimized clinical management.

**Methods:**

We searched PubMed, Embase, Cochrane Library, Web of Science, and Scopus up to August 16, 2026. Two reviewers independently performed study screening and data extraction. Risk of bias was assessed using RoB 2. Meta-analyses were conducted in Stata 18.0. For heterogeneous outcomes, 95% prediction intervals (PIs) were calculated to estimate effect ranges for future studies. Heterogeneity was explored via leave-one-out sensitivity, meta-regression, and subgroup analyses. The GRADE (Grading of Recommendations Assessment, Development, and Evaluation) framework rated evidence certainty. Publication bias was evaluated for outcomes reported in ≥10 studies.

**Results:**

Twenty-three RCTs (3055 women with GDM) were included. Pooled estimates suggested that telemedicine may reduce fasting glucose (mean difference [MD] −0.26, 95% CI −0.42 to −0.09, 95% PI −0.93 to 0.42; *P*=.002), 2-hour postprandial glucose (MD −0.50, 95% CI −0.87 to −0.14, 95% PI −1.84 to 0.84; *P*=.007), and gestational weight gain (MD −0.78, 95% CI −1.40 to −0.17, 95% PI −2.83 to 1.26; *P*=.01). Telemedicine was also associated with lower risks of cesarean section, emergency cesarean section, macrosomia, neonatal hypoglycemia, and neonatal respiratory distress. No significant differences were found for hemoglobin A_1c_, preterm birth, or neonatal intensive care unit admission. Notably, fasting glucose, 2-hour postprandial glucose, and gestational weight gain exhibited very high unexplained heterogeneity (*I*²>90%), with their 95% PIs crossing the null value despite statistically significant pooled effects. The GRADE certainty of evidence was very low to low across all assessed outcomes.

**Conclusions:**

Telemedicine may improve selected glycemic and perinatal outcomes for women with GDM and may serve as a promising supplementary option for prenatal care, particularly in resource-limited settings where routine in-person follow-up is difficult to implement. Nevertheless, the apparent benefits for glycemic control and gestational weight gain remain tentative due to extreme, unexplained between-study heterogeneity and 95% PIs crossing the null. Based on current evidence, telemedicine should not completely replace standard antenatal care. Future well-designed, large-sample RCTs are warranted; such trials should adopt standardized definitions for telemedicine interventions, fully report intervention components, and conduct long-term follow-up to verify sustained maternal and neonatal clinical benefits.

## Introduction

Gestational diabetes mellitus (GDM) is currently the most common medical complication of pregnancy, defined as “any degree of glucose intolerance that occurs during pregnancy or is first detected during pregnancy” [1,2]. The diagnosis of GDM typically occurs in weeks 24 to 28 of pregnancy, when routine oral glucose tolerance test screening is performed [3]. Its core pathophysiological features include increased insulin resistance and insufficient compensatory function of pancreatic beta cells [4]. The incidence of GDM varies by region and diagnostic criteria. According to the 2021 International Diabetes Federation Diabetes Atlas, GDM affects 14.0% of pregnancies worldwide, and the incidence rate is still on the rise [5,6]. GDM not only has adverse effects on the health of pregnant women [1] but also increases the risks of adverse pregnancy outcomes such as preterm birth, macrosomia, neonatal hypoglycemia, and respiratory distress syndrome [7]. Although GDM usually resolves on its own after childbirth, its potential long-term effects include, but are not limited to, a risk of developing type 2 diabetes within 5 to 10 years, higher risks of developing cardiovascular disease for mothers [8,9], and higher rates of obesity, late-onset diabetes, and cardiovascular disease in adulthood for infants [10-12].

The aim of GDM treatment and patient education is to maintain glucose levels within the recommended glycemic reference range to prevent maternal hyperglycemia or hypoglycemia [13,14]. However, the traditional GDM management approach has several drawbacks, including discontinuous data, delayed intervention, limited personalization, and poor compliance [15-17]. Technological innovations present exciting opportunities for novel approaches to improve clinical care delivery for women with diabetes during pregnancy. Telemedicine is a type of medical and health service that is carried out through communication technologies such as mobile phones and the internet. Medical staff can use systems based on websites or mobile terminal devices to assess, diagnose, and treat patients, monitor health indicators in real time, and provide timely feedback [18]. They can also disseminate health knowledge and simultaneously offer professional guidance to improve the physical and mental health conditions of patients [19,20]. The telemedicine platform has facilitated real-time communication beyond the limitations of time and space, enabling pregnant women to promptly report symptoms and seek advice [21]. This has promoted a shift in the interaction model between patients and providers from “passive follow-up” to “active management.” At the same time, medical professionals customize diet and exercise plans based on gestational weeks, dietary and lifestyle habits, and blood sugar data, leading to the development of intervention methods from “standardization” to “personalization” [22,23].

In recent years, telemedicine has garnered significant attention in health care and has been widely integrated into health systems. Research shows that telemedicine can effectively improve the physical and mental conditions of patients with cancer, patients with stroke, and those with chronic diseases, enhancing the effectiveness of disease management and the quality of life [24-26]. Nevertheless, the available synthesized evidence on the efficacy of telemedicine specifically for GDM remains limited and inconsistent. Several prior systematic reviews exploring telemedicine for GDM have notable limitations. An early meta-analysis by Rasekaba et al [27] included only a small number of older randomized controlled trials (RCTs). Ming et al [19] pooled mixed cohorts of pregestational and gestational diabetes rather than focusing purely on GDM. Xie et al [18] included a large set of RCTs but lacked a formal GRADE (Grading of Recommendations Assessment, Development, and Evaluation) evidence-certainty evaluation. Bertini et al [28] provided only a qualitative narrative review without a quantitative meta-analysis, while Yang et al [21] restricted their analysis exclusively to mobile health tools and omitted broader telemedicine modalities.

Building on these gaps, the present meta-analysis adds several key contributions compared with earlier work. First, we strictly focus on GDM-only participants and cover the full range of telemedicine modalities for stratified subgroup analysis. Second, we incorporate underassessed end points, including emergency cesarean section and gestational weight gain, alongside individual neonatal outcomes such as macrosomia, neonatal hypoglycemia, and neonatal respiratory distress, to obtain a more comprehensive perinatal risk profile. Third, beyond standard heterogeneity statistics, we apply 95% prediction intervals (PIs), meta-regression, and modality-based subgroup analysis to evaluate the generalizability of results. Fourth, we apply GRADE to rate the certainty of evidence and cautiously interpret heterogeneous pooled estimates instead of overstating findings driven solely by statistical significance.

To address these gaps in the literature, we conducted this systematic review and meta-analysis. Our objectives were to evaluate telemedicine-related maternal-fetal outcomes in women with GDM, quantify the pooled effect magnitude and consistency, apply GRADE to rate the certainty of evidence, and provide actionable insights for future digital-health RCTs.

## Methods

### Overview

This systematic review and meta-analysis adheres to the PRISMA (Preferred Reporting Items for Systematic Reviews and Meta-Analyses) 2020 reporting guidelines [29], with the completed checklist available in Checklist 1. This review was retrospectively registered on PROSPERO (International Prospective Register of Systematic Reviews; CRD420261403669). The initially submitted version 1.0 of the PROSPERO protocol contained several discrepancies relative to the actual review workflow; these deviations have been formally amended and updated within the PROSPERO registry record. All protocol modifications are documented and explained in Multimedia Appendix 1.

### Search Strategy

A comprehensive electronic literature search was conducted across PubMed, Embase, Cochrane Library, Web of Science, and Scopus from the inception of each database up to August 16, 2026. The search strategy incorporated both MeSH and free-text terms. Full search strings for each database are provided in Multimedia Appendix 2. Manual reference-list screening of included papers and relevant systematic reviews was performed to retrieve additional eligible records.

### Inclusion and Exclusion Criteria

We established inclusion and exclusion criteria based on PICOS (Population, Interventions, Comparison, Outcomes, and Study type) questions. The inclusion criteria encompassed the following aspects:

1. Population: individuals officially diagnosed with GDM
2. Interventions: telehealth or telemedicine interventions delivered via the internet, websites, telephone calls, apps, SMS text messages, or other digital communication technologies
3. Comparison: usual care, routine care, conventional care, or standard care without telemedicine
4. Study type: RCTs

The following criteria were used for exclusion:

1. Studies published in non–English languages
2. Studies lacking a non-telemedicine comparator arm or lacking a telemedicine intervention
3. Review papers, protocols, ongoing studies, and duplicate publications
4. Studies lacking statistical data for analysis

### Study Selection

All retrieved records from the databases were imported into EndNote X9 software (Clarivate Analytics) for deduplication and literature management. Two independent reviewers (HH and JD) initially screened titles and abstracts based on predeﬁned inclusion criteria. For studies that initially met the criteria, full texts were retrieved and further screened to determine final inclusion. Reasons for excluding full-text papers were documented for the PRISMA flow diagram. Any disagreements between the two reviewers during the screening process were resolved through discussion; when necessary, a third reviewer (JL) was consulted to provide arbitration.

### Data Extraction

This study strictly adhered to the PRISMA statement for data extraction to ensure methodological systematicity. Two reviewers (HH and JD) independently extracted data using a pretested data extraction form, and a third author (XX) cross-verified the accuracy of the results. The extracted data included authors, country, year of publication, population characteristics (age, gestational age at enrollment, and baseline glucose levels), study duration, intervention methods in both the intervention and control groups, and study outcomes (fasting blood glucose, 2-h postprandial blood glucose, hemoglobin A_1c_ [HbA_1c_], gestational weight gain, cesarean section, emergency cesarean section, preterm birth, macrosomia, neonatal hypoglycemia, neonatal respiratory distress, admission to the neonatal intensive care unit [NICU]).

### Quality Assessment

Two reviewers (HH and JD) independently performed the quality assessment. Any assessment discrepancies were resolved through discussion with a third reviewer (JC) until consensus was reached. The risk of bias was evaluated using the revised Cochrane Risk of Bias tool (RoB 2), focusing on the following 5 domains: the randomization process, deviations from intended interventions, missing outcome data, outcome measurement, and selection of the reported result [30].

### Data Synthesis and Analysis

All statistical analyses were performed using Stata 18.0 (StataCorp). For continuous outcomes, effect sizes were expressed as mean difference (MD) with a 95% CI. MD quantifies the absolute disparity between the mean values of the treatment and control arms, representing the net change in the outcome attributable to the intervention. For dichotomous outcomes, risk ratio (RR) with a 95% CI was employed as the summary statistic. RR is defined as the likelihood ratio of an event occurring between the experimental and comparator groups, reflecting the relative increase or decrease in risk conferred by the intervention. A smaller RR value corresponds to a greater reduction in event risk, indicating more favorable outcomes for the intervention group.

Heterogeneity across studies was assessed using the Cochran Q test and quantified by the *I*² statistic. *I*²>40% was considered to indicate substantial heterogeneity, prompting the use of a random effects model; otherwise, a fixed effects model was applied. For outcomes with substantial heterogeneity (*I*²>40%), we explored potential sources through sensitivity analysis, meta-regression, and subgroup analyses based on predefined study-level characteristics [31]. For outcomes with substantial heterogeneity, we calculated the 95% PI to estimate the range in which the true effect of a future comparable study would be expected to lie [32]. The 95% PI accounts for both the uncertainty of the pooled estimate and between-study heterogeneity, thereby providing a clinically meaningful range of expected effects across settings. A 95% PI that includes the null value indicates that the intervention may not confer benefit in all future populations, even when the pooled effect is statistically significant. Publication bias was assessed via funnel plots and the Egger regression test only when ≥10 studies were available for an outcome [33]. All statistical tests were 2-sided, and a *P* value <.05 was considered statistically significant for the overall effect size. The certainty of evidence for each outcome was evaluated using the GRADE approach (Multimedia Appendix 3) [34]. The GRADE approach is a widely recognized tool for grading the certainty of evidence in systematic reviews and clinical practice guidelines, classifying certainty into 4 levels: “very low,” “low,” “moderate,” and “high,” with each level determined according to specific criteria. Factors that may lower the certainty of evidence (such as risk of bias, inconsistency, indirectness, imprecision, and publication bias) were assessed, as were factors that may raise this certainty (such as a large magnitude of effect, plausible residual confounding, and dose-response relationships).

## Results

### Study Selection

A total of 2777 records were retrieved from 5 databases. After removing 1153 duplicate records using EndNote X9, 1624 papers remained. Following title-and-abstract screening, full texts of 166 potentially eligible records were retrieved for further assessment. After full-text evaluation, 23 RCTs met the predefined inclusion criteria and were included in this systematic review and meta-analysis [22,35-56]. The detailed study selection process and reasons for exclusion are illustrated in Figure 1.

**Figure 1.**
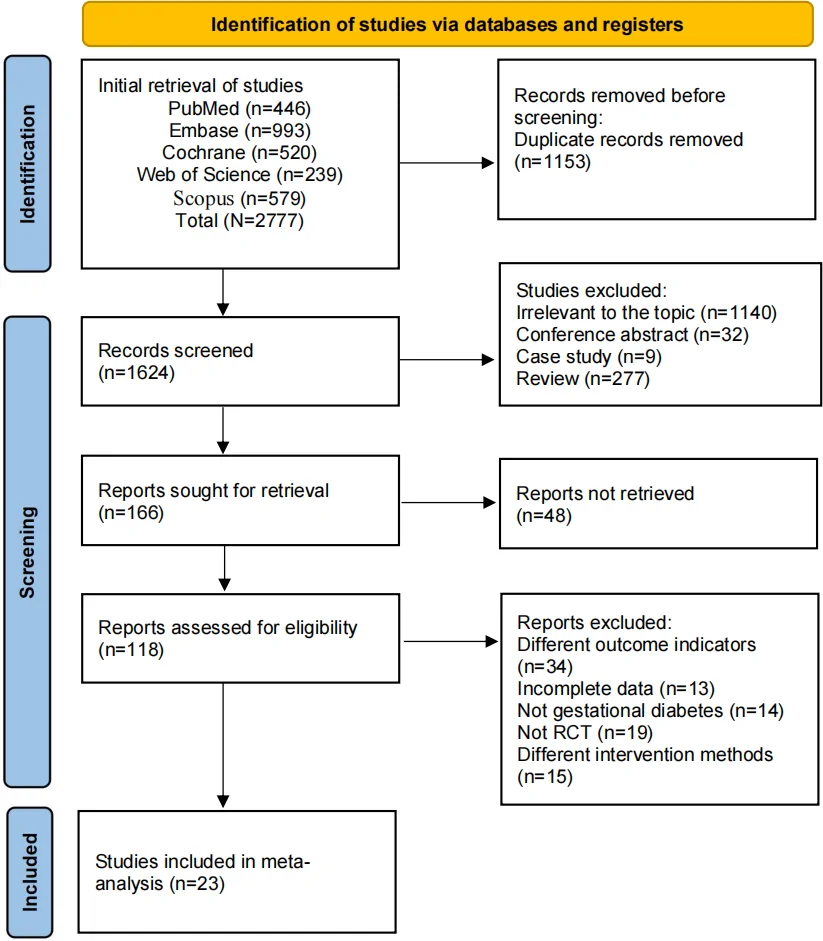
PRISMA (Preferred Reporting Items for Systematic Reviews and Meta‑Analyses) flow diagram of the literature screening and selection process. RCT: randomized controlled trial.

### Study Characteristics

A total of 23 RCTs involving 3055 participants were included. Publication years of included studies spanned from 2007 to 2026. Studies were conducted across multiple countries and regions, including Asia (Korea [35], Malaysia [36], China [38,43,47-50,55], Japan [39], Turkey [41], Iran [42,45], Singapore [46], and Israel [53]), North America (United States [37,54,56]), Europe (United Kingdom [40], Finland [22], Slovenia [44], and Norway [51]), and Oceania (Australia [52]). Total sample sizes per individual study ranged from 21 to 350 participants. Mean participant age was reported in 20 studies [22,35-48,50,52-54,56] and ranged from 29.5 to 36.4 years. Gestational age at enrollment was available for 13 studies [22,35,37,40,43-47,50,52,54,56], varying between 19.5 and 31.0 weeks. Baseline fasting glucose values were reported in 15 studies [22,35,37-40,43,44,46-48,52-54,56], ranging from 4.46 to 5.39  mmol/L. With respect to intervention duration, the telemedicine intervention continued until delivery in 12 studies [22,35,37,38,40,44,46,47,49,53-55], while the remaining 11 studies [36,39,41-43,45,48,50-52,56] reported specific intervention durations. Telemedicine interventions were grouped into 3 primary modalities: health applications, website-based platforms, and WeChat-based platforms. A small subset of studies [36,37,45,54] adopted combined approaches incorporating text message or telephone-based support, or interactive voice response functions. Full characteristics of the included studies are presented in Table 1.

**Table 1.** Characteristics of included studies.

Study and year	Country	Sample		Mean age (y)	Gestational age at enrollment (wk)	Baseline fasting glucose (mmol/L)	Intervention duration	Intervention methods		Main outcomes^a^
		Intervention group	Control group					Intervention group	Control group	
Sung et al [35] (2019)	Korea	11	10	33.43	27.33	5.11	To birth	Health app	Usual care	①④⑤
Benton et al [36] (2026)	Malaysia	30	30	31.3	—^b^	—	6 months after delivery	Health app + text	Usual care	①③
Ying and Ouyang [37] (2021)	United States	40	40	31.0	28.45	5.36	To birth	Web-based system + IVR^c^	Usual care	①⑤⑦⑨⑩⑪
Wang et al [38] (2026)	China	47	47	32.18	—	5.39	To birth	WeChat	Usual care	①②③④⑦⑧
Aoyama et al [39] (2026)	Japan	18	20	36.4	—	5.07	10 weeks	Health app	Usual care	③④⑤⑥⑧⑨⑪
Mackillop et al [40] (2018)	United Kingdom	101	102	33.4	31.0	5.2	To birth	Health app	Usual care	④⑤⑥⑦⑨
Akbag and Tokat [41] (2026)	Turkey	31	34	31.2	—	—	4‐12 weeks after delivery	Web-based system	Usual care	①⑧⑪
Salarkarimi et al [42] (2025)	Iran	40	40	31.2	—	—	10 weeks	Health app	Usual care	①②
Pan et al [43] (2025)	China	85	86	31.2	26.3	5.0	12 weeks	WeChat	Usual care	①②⑧
Kytö et al [22] (2024)	Finland	76	72	34.1	26.4	5.08	To birth	Health app	Usual care	①④⑤⑥⑦⑧⑨⑩⑪
Munda et al [44] (2023)	Slovenia	53	52	32.4	19.5	5.10	To birth	Health app	Usual care	①③④⑤⑦
Maleki et al [45] (2023)	Iran	38	38	29.7	26.6	—	4 weeks	Health app + Telephone/text	Usual care	①②
Yew et al [46] (2021)	Singapore	170	170	32.0	26.9	4.7	To birth	Health app	Usual care	④⑤⑥⑦⑧⑨⑩⑪
Tian et al [47] (2021)	China	136	133	31.07	26.37	4.98	To birth	WeChat	Usual care	⑤⑦⑧
Su et al [48] (2021)	China	56	56	35.8	—	4.46	6 months	Health app	Usual care	①④⑤⑦⑧⑪
Guo et al [49] (2021)	China	70	70	—	—	—	To birth	WeChat	Usual care	①②⑦⑧⑩
Guo et al [50] (2019)	China	64	60	30.91	24.89	—	13 weeks	Health app	Usual care	①②③④⑤⑧⑨
Borgen et al [51] (2019)	Norway	112	121	—	—	—	3 months after delivery	Health app	Usual care	②⑤⑥⑧⑪
Rasekaba et al [52] (2018)	Australia	61	34	32.0	28.0	5.16	10 weeks	Web-based system	Usual care	⑤⑥⑧⑪
Miremberg et al [53] (2018)	Israel	60	60	31.9	—	4.90	To birth	Health app	Usual care	⑤⑥⑨⑩⑪
Homko et al [54] (2012)	United States	36	38	30.2	28.5	5.36	To birth	Web-based system + IVR	Usual care	①②⑤⑦⑨⑩⑪
Zhou and Gan [55] (2023)	China	182	168	—	—	—	To birth	WeChat	Usual care	①②③④⑤⑦⑧⑨
Homko et al [56] (2007)	United States	32	25	29.5	27.6	5.08	8 weeks	Web-based system	Usual care	①②③⑤⑦⑨⑩⑪

a ① Fasting blood glucose; ② 2-hour postprandial blood glucose; ③ hemoglobin A_1c_; ④ gestational weight gain; ⑤ cesarean section; ⑥ emergency cesarean section; ⑦ preterm birth; ⑧ macrosomia; ⑨ neonatal hypoglycemia; ⑩ neonatal respiratory distress; ⑪ admission to the neonatal intensive care unit.

b Not available.

c IVR: interactive voice response.

### Quality Assessment Results of the Studies

Risk-of-bias evaluation of the 23 included RCTs was performed using RoB 2. Overall, 6 studies were judged to have low risk of bias, 7 studies had high risk of bias, and 10 studies raised some concerns for risk of bias. The most frequent source of bias was missing outcome data, driven by substantial participant attrition and an absence of appropriate statistical correction in most publications. The summary risk-of-bias judgments are presented in Figure 2.

**Figure 2.**
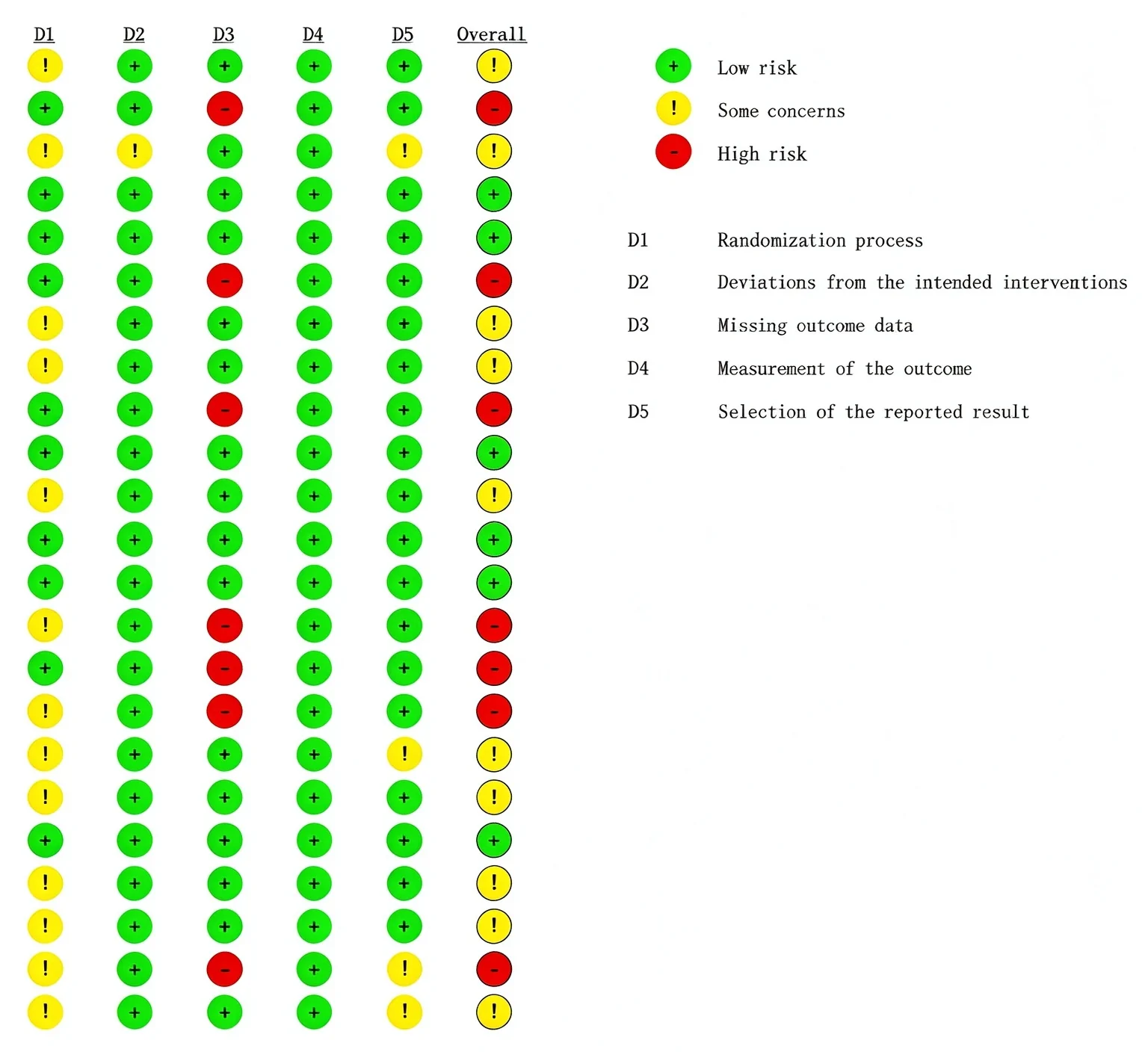
Assessment of risk of bias. RoB 2: revised Cochrane risk-of-bias tool for randomized trials.

### Meta-Analysis Results

#### Effects on Blood Glucose

The meta-analysis of 16 trials [22,35-38,41-45,48-50,54-56] using a random effects model showed that fasting glucose was significantly lower in the telemedicine group compared with the control group (MD −0.26, 95% CI −0.42 to −0.09, 95% PI −0.93 to 0.42; *P*=.002), with high heterogeneity (*I*²=94.1%, *P*<.001). The forest plots are shown in Figure 3. The 95% PI crossed the null value, indicating that the true effect in future comparable clinical settings might be null or might favor usual care. Sensitivity analysis showed minimal changes in the pooled effect and *I*² after item-by-item exclusion. Meta-regression and subgroup analyses based on publication year, country of publication, and gestational age at enrollment did not identify a convincing source of this substantial heterogeneity. Therefore, the observed inconsistency remains largely unexplained.

A meta-analysis of 10 trials [38,42,43,45,49-51,54-56] using a random effects model showed that the 2-hour postprandial glucose level was significantly lower in the telemedicine group than in the control group (MD −0.50, 95% CI −0.87 to −0.14, 95% PI −1.84 to 0.84; *P*=.007), with high heterogeneity (*I*²=91.3%, *P*<.001). The 95% PI also crossed the null value. The forest plots are shown in Figure 4. Sensitivity analysis revealed that the pooled effect size and *I*² changed minimally after sequentially excluding each study. Meta-regression and subgroup analyses based on publication year, country of publication, and gestational age at enrollment did not explain the substantial heterogeneity for 2-hour postprandial glucose.

A total of 7 studies [36,38,39,44,50,55,56] were included in the meta-analysis. Due to substantial heterogeneity across studies (*I*²=96.7%, *P*<.001), a random effects model was applied. Pooled results suggested that the telemedicine intervention was associated with a reduction in HbA_1c_ relative to usual care; however, the difference did not reach statistical significance (MD −0.29, 95% CI −0.64 to 0.07, 95% PI −1.54 to 0.97; *P*=.11).

**Figure 3.**
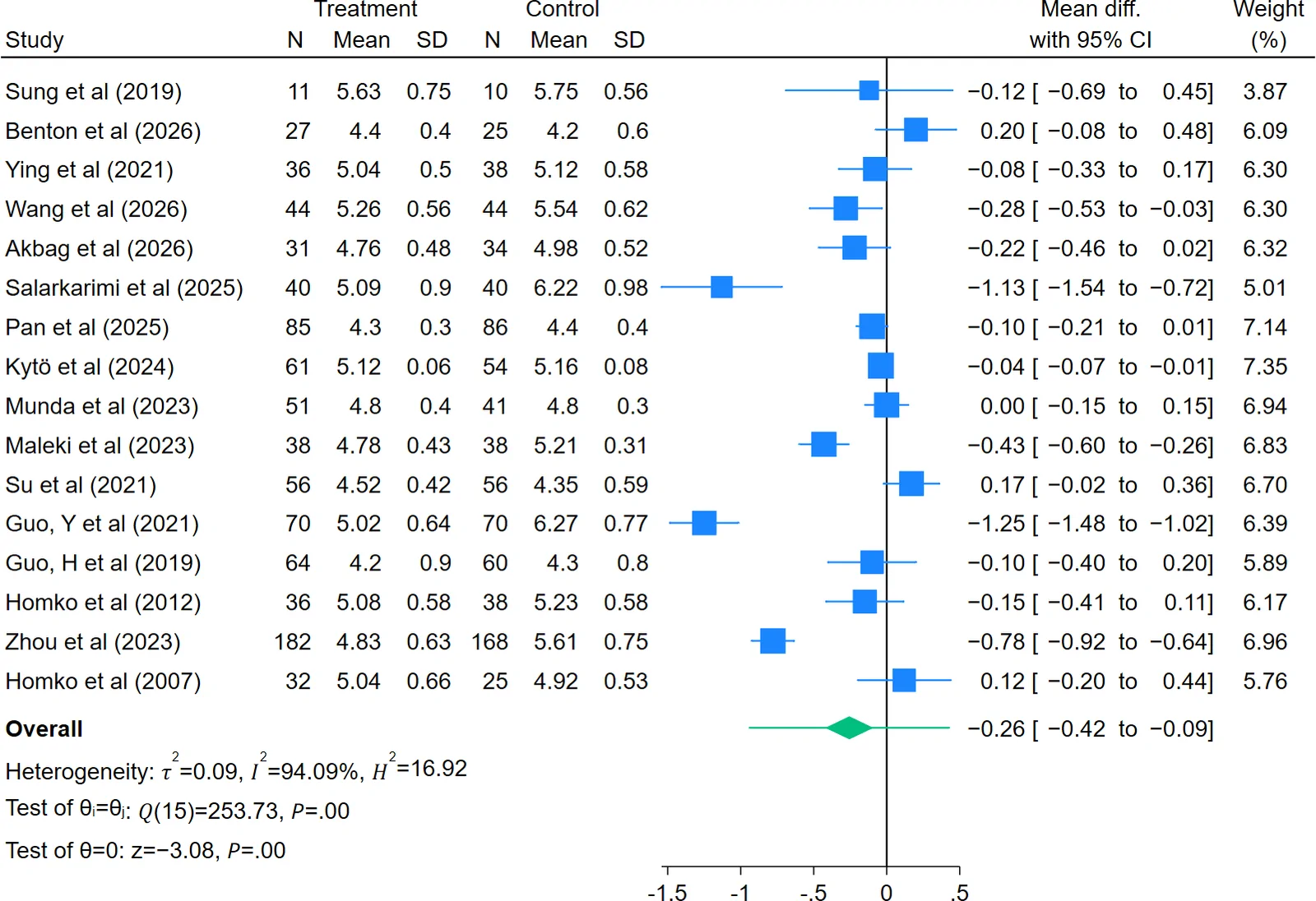
Effect of telemedicine on blood glucose. Weights are calculated from the random effects model [22,35-38,41-45,48-50,54-56].

**Figure 4.**
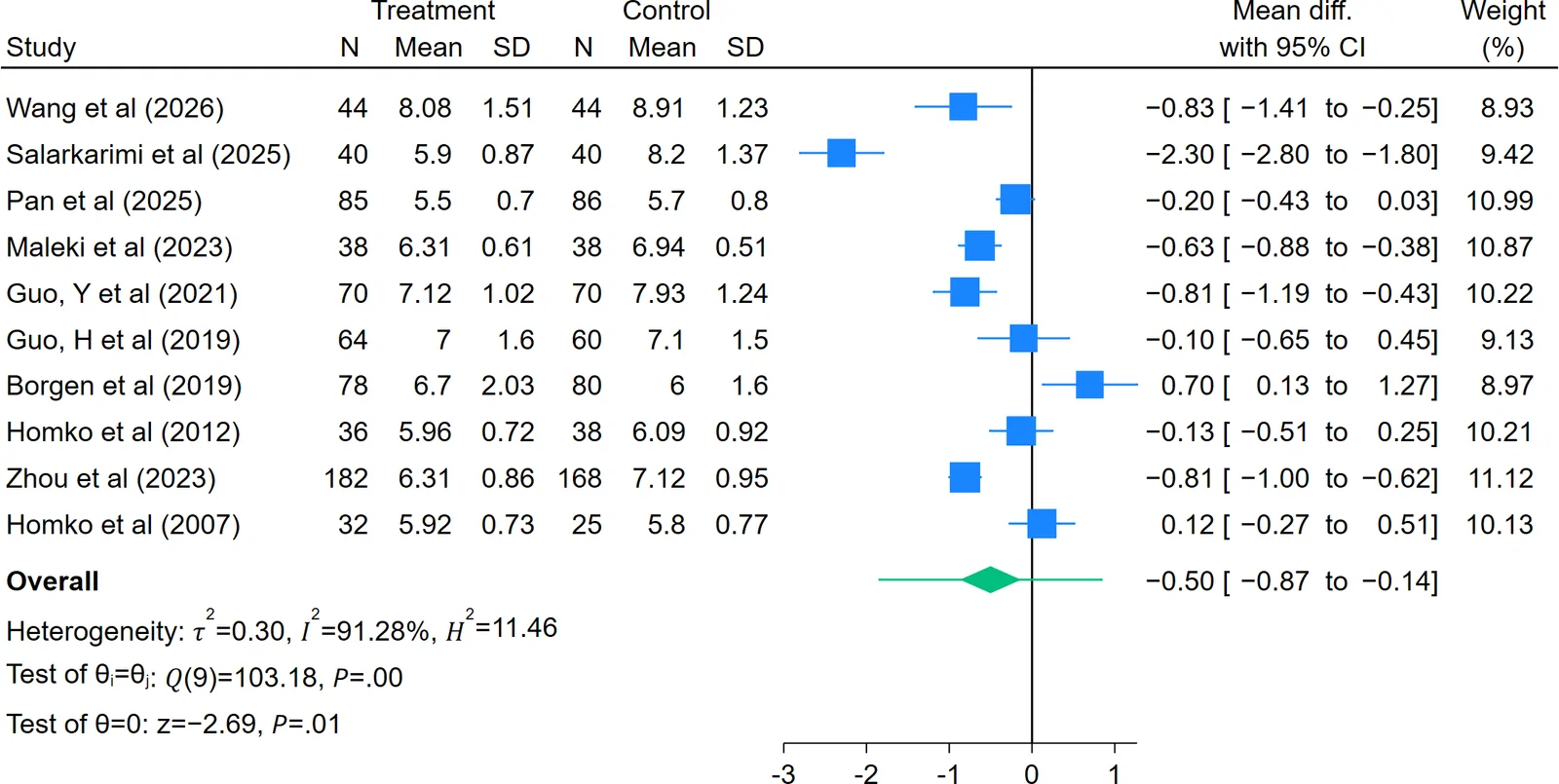
Effect of telemedicine on 2-hour postprandial blood glucose. Weights are calculated from the random effects model [38,42,43,45,49-51,54-56].

#### Effects on Gestational Weight Gain

A meta-analysis of 10 trials [22,35,38-40,44,46,48,50,55] using a random effects model showed that gestational weight gain was significantly lower in the telemedicine group compared with the control group (MD −0.78, 95% CI −1.39 to −0.16, 95% PI −2.81 to 1.26; *P*=.01), with high heterogeneity (*I*²=90.3%, *P*<.001). The 95% PI crossed the null value. The forest plots are shown in Figure 5. Sensitivity analysis showed that after excluding Guo et al [50], *I*² decreased from 90.3% to 77.5%, while the overall effect remained robust (MD −0.61, 95% CI −1.16 to −0.07, 95% PI −2.22 to 1.00; *P*=.03), suggesting that this study contributed substantially to the observed heterogeneity. However, even after this exclusion, substantial heterogeneity persisted. Meta-regression and subgroup analyses did not identify a convincing source of the inconsistency. The pooled estimate for gestational weight gain should therefore be interpreted with caution.

**Figure 5.**
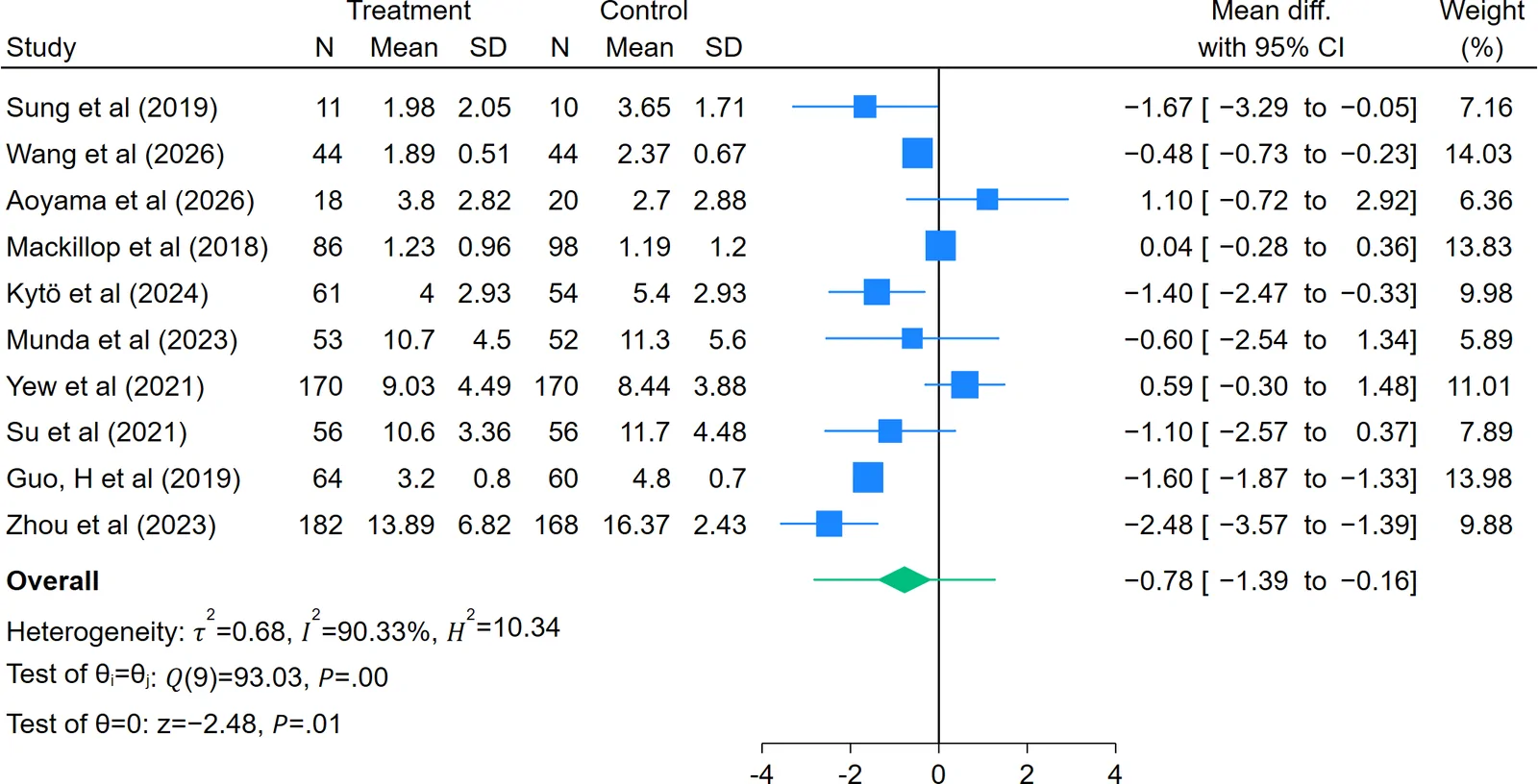
Effect of telemedicine on gestational weight gain. Weights are calculated from the random effects model [22,35,38-40,44,46,48,50,55].

#### Effects on Delivery Mode

A random effects model was used to evaluate cesarean section outcomes across 16 trials [22,35,37,39,40,44,46-48,50-56]. The pooled analysis showed that telemedicine intervention had a significant effect in reducing the incidence of cesarean section (RR 0.84, 95% CI 0.72 to 0.996, 95% PI= −0.51 to 1.40; *P*=.045), with moderate heterogeneity among studies (*I*²=46.7%, *P*=.02). The forest plots are shown in Figure 6. Sensitivity analysis revealed that the trial by Homko et al [56], which targeted high-risk, low-income pregnant women with poor adherence to remote monitoring, contributed substantially to between-study heterogeneity. After excluding this trial, telemedicine demonstrated a significant protective effect in reducing the cesarean section rate (RR 0.81, 95% CI 0.70-0.95, 95% PI 0.54-1.22; *P*=.008), and heterogeneity was substantially reduced (*I*²=34.5%, *P*=.09).

A meta-analysis of 7 trials [22,39,40,46,51-53] using a fixed effects model showed that the incidence of emergency cesarean section was significantly lower in the telemedicine group than in the control group (RR 0.59, 95% CI 0.45-0.79; *P*<.001), with no significant heterogeneity among studies (*I*²=36.8%, *P*=.15). The forest plots are shown in Figure 7.

**Figure 6.**
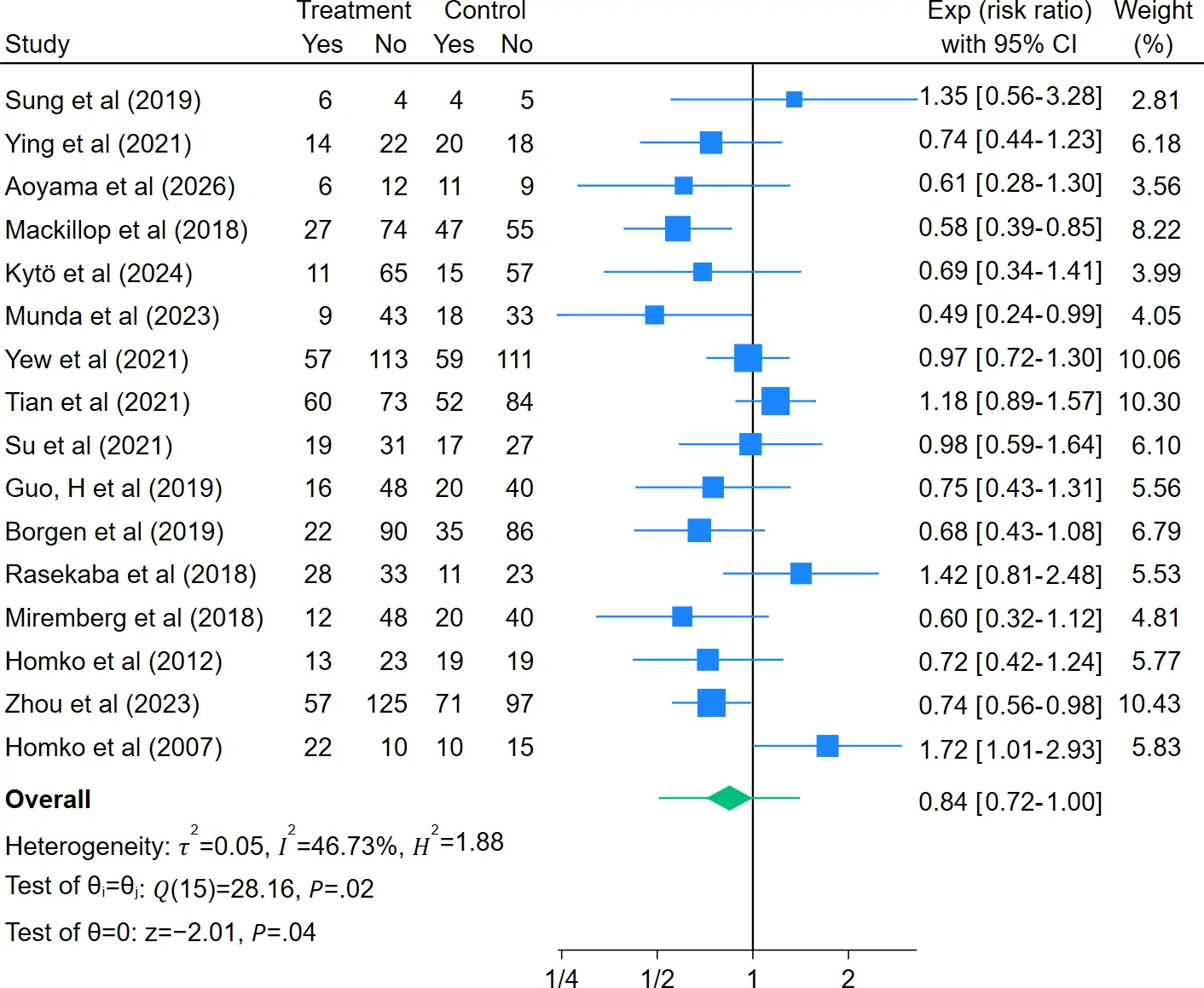
Effect of telemedicine on cesarean section. Weights are calculated from the random effects model [22,35,37,39,40,44,46-48,50-56].

**Figure 7.**
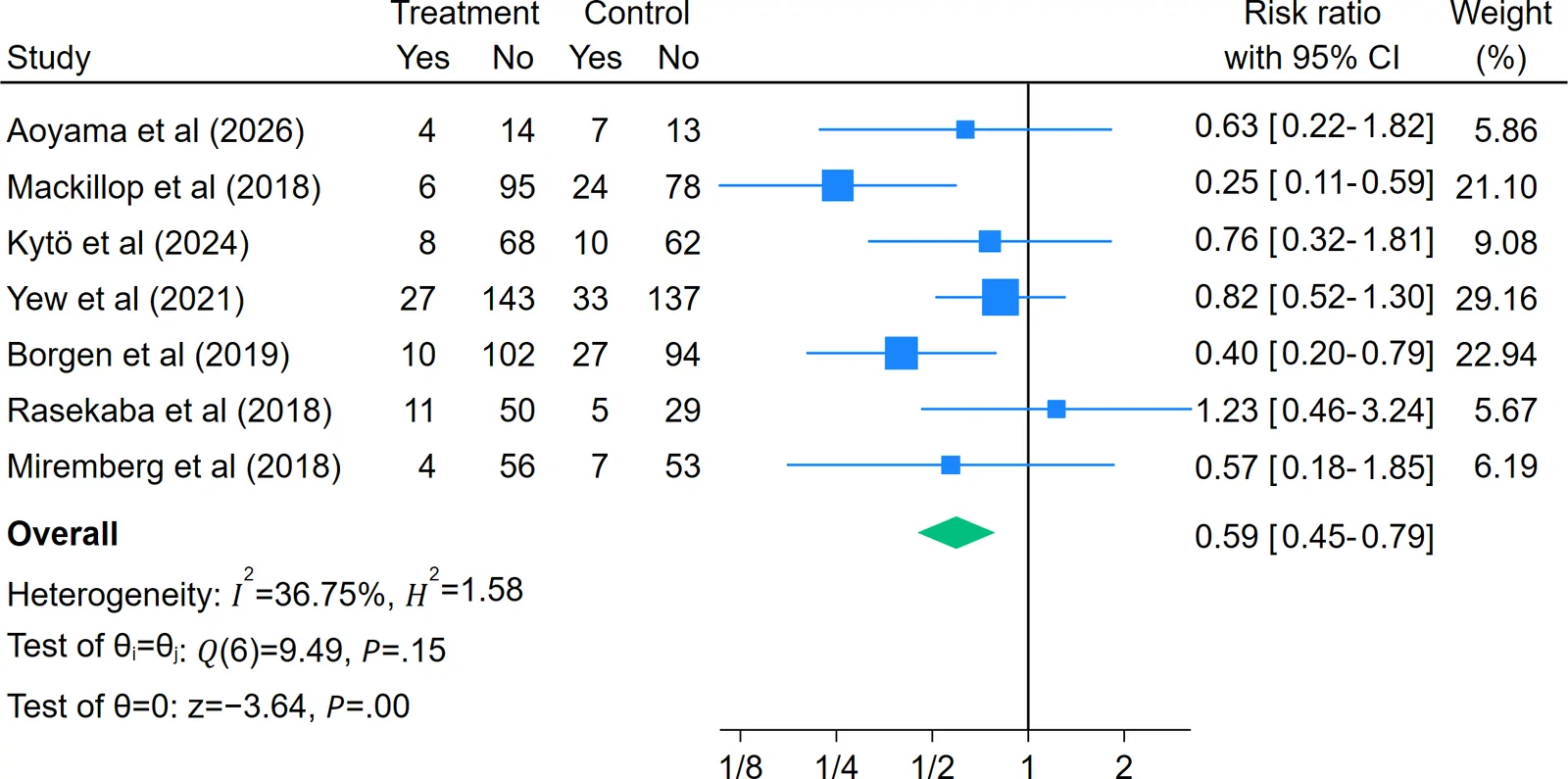
Effect of telemedicine on emergency cesarean section. Weights are calculated from the random effects model [22,39,40,46,51-53].

#### Effects on Unfavorable Pregnancy Outcomes

A total of 12 studies [22,37,38,40,44,46-49,54-56] were pooled using a fixed effects model, showing low-to-moderate heterogeneity (*I*²=30.4%, *P*=.15). Compared with the usual care group, the telemedicine intervention group showed no statistically significant difference in the risk of preterm birth (RR 0.76, 95% CI 0.57-1.01; *P*=.06). To test the robustness of the results, we also performed a sensitivity analysis using a random effects model. The results showed a pooled RR of 0.78 (95% CI 0.54-1.12; *P*=.18). Findings were largely consistent across models, indicating the results were robust to the choice of statistical model.

The pooled analysis of 12 studies [22,38,41,43,46-52,55] using a fixed effects model demonstrated a significantly lower incidence of macrosomia in the telemedicine group relative to the control group (RR 0.56, 95% CI 0.42-0.74; *P*<.001; *I*²=0%, *P*=.65). The forest plots are shown in Figure 8.

**Figure 8.**
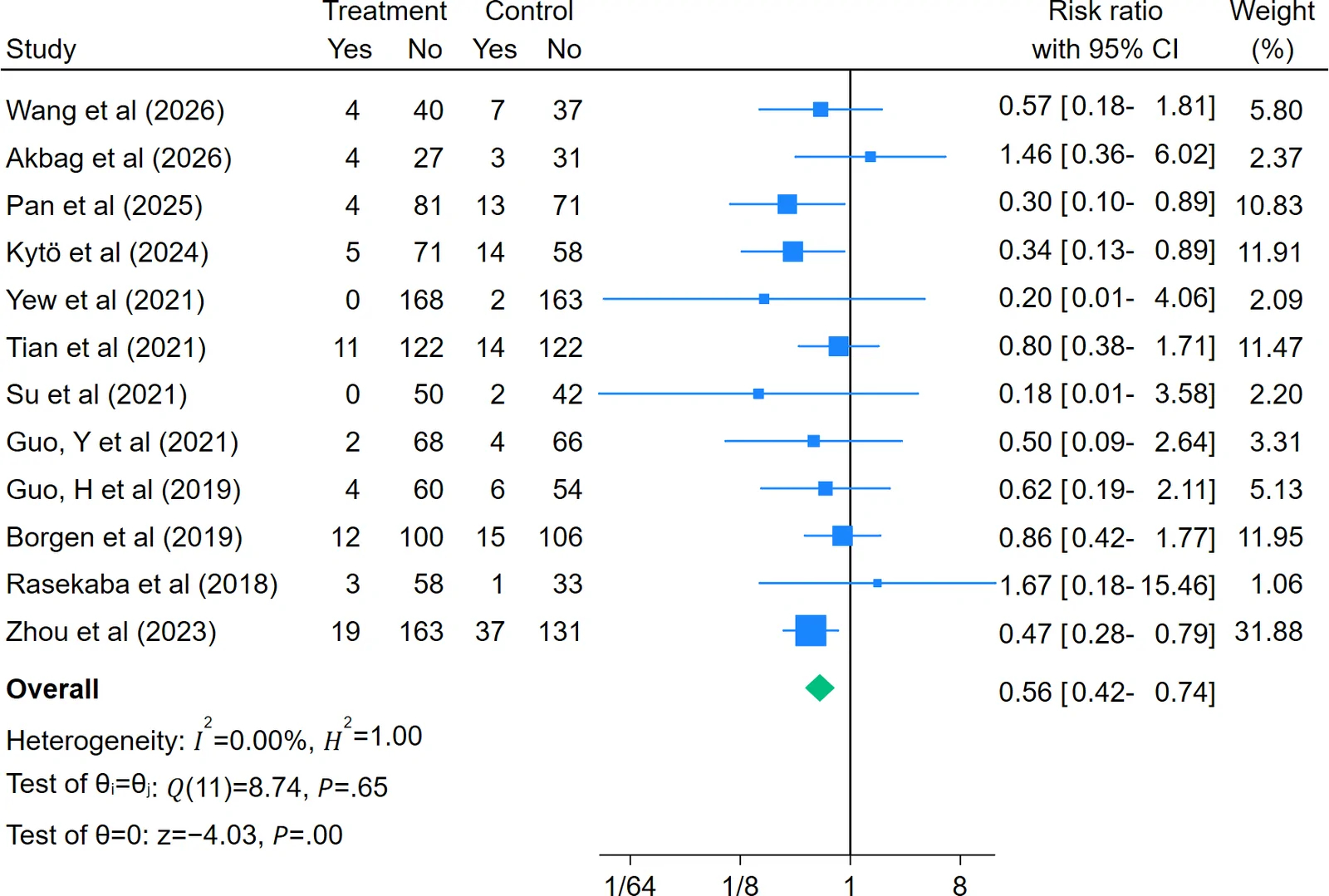
Effect of telemedicine on macrosomia. Weights are calculated from the random effects model [22,38,41,43,46-52,55].

#### Effects on Neonatal Complications

A meta-analysis of 10 studies [22,37,39,40,46,50,53-56] using a fixed effects model showed a significantly lower risk of neonatal hypoglycemia in the telemedicine group relative to usual care (RR 0.77, 95% CI 0.61-0.98; *P*=.03), with low-to-moderate heterogeneity (*I*²=29.8%, *P*=.17). The forest plots are shown in Figure 9.

A meta-analysis of 7 studies [22,37,46,49,53,54,56] using a fixed effects model showed a significantly lower risk of neonatal respiratory distress in the telemedicine group relative to usual care (RR 0.65, 95% CI 0.46-0.92; *P*=.02), with low heterogeneity (*I*²=7.2%, *P*=.37). The forest plots are shown in Figure 10.

**Figure 9.**
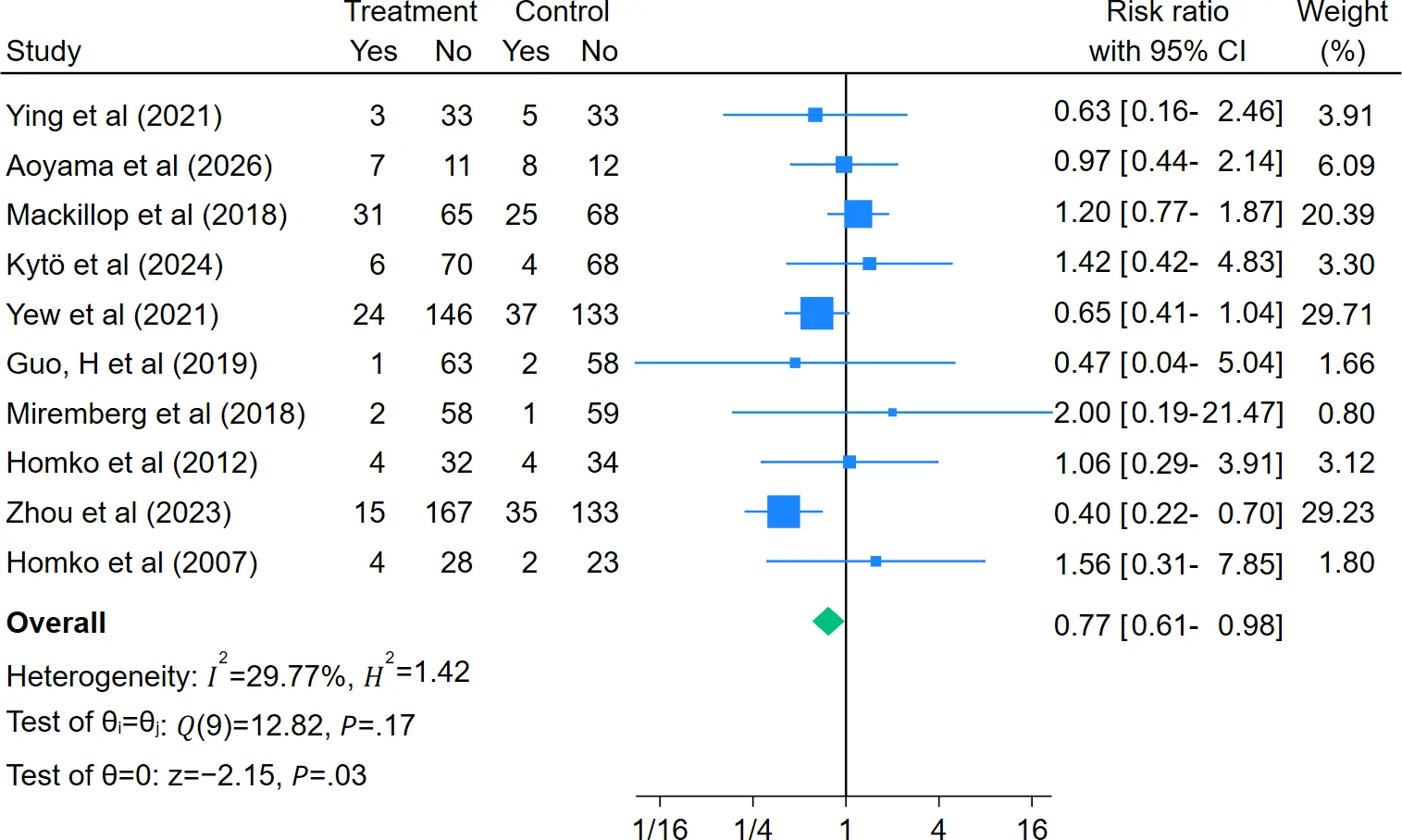
Effect of telemedicine on neonatal hypoglycemia. Weights are calculated from the random effects model [22,37,39,40,46,50,53-56].

**Figure 10.**
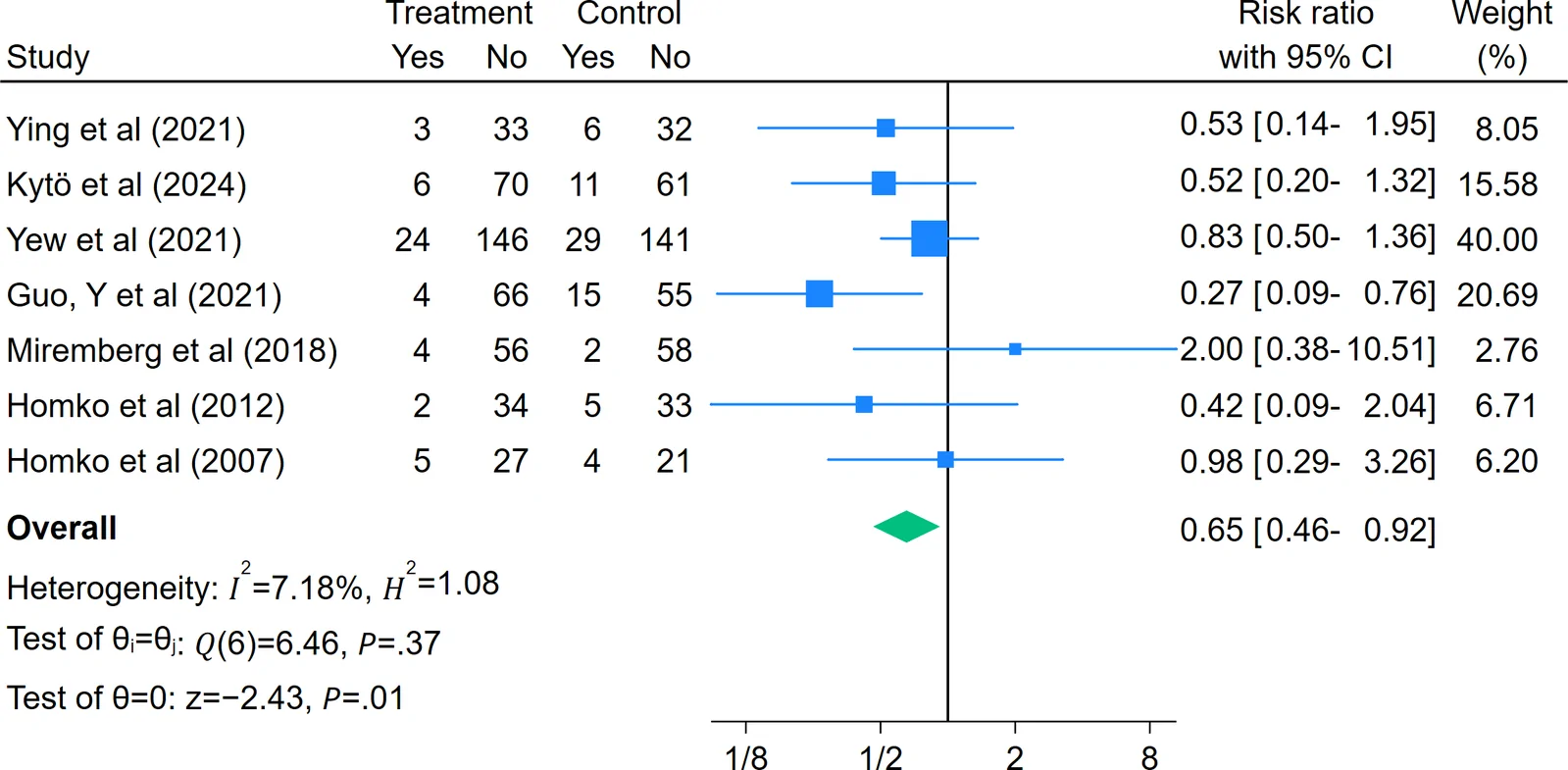
Effect of telemedicine on neonatal respiratory distress. Weights are calculated from the random effects model [22,37,46,49,53,54,56].

A meta-analysis of 11 studies [22,37,39,41,46,48,51-54,56] using a fixed effects model found no significant difference in the risk of admission to the NICU between the telemedicine group and the usual-care group (RR 0.83, 95% CI 0.62-1.11; *P*=.21), with low heterogeneity (*I*²=0%, *P*=.72).

### Subgroup Analysis

To explore potential sources of substantial heterogeneity in fasting glucose, 2-hour postprandial blood glucose, and gestational weight gain, we performed subgroup analyses stratified by publication year (≤2020 and >2020), publication region (Asia, North America, Europe, and Oceania), and intervention modality (health applications, web-based platforms, and WeChat-based platforms). The results of the subgroup analyses are presented in Table 2.

**Table 2.** Subgroup analysis.

Outcome indicators	Number of studies	Heterogeneity test		Meta-analysis		
		*I*^2^ (%)	*P* value	MD^a^ (95% CI)	*P* value	95% PI^b^
Fasting blood glucose						
Publication year						
≤2020	4	0	.60	−0.06 (−0.22 to 0.10)	.46	(−0.41 to 0.29)
>2020	12	95.6	<.001	−0.31 (−0.50 to −0.12)	.002	(−1.06 to 0.44)
Publication country						
Asia	11	94.2	<.001	−0.37 (−0.63 to −0.10)	.007	(−1.37 to 0.64)
North America	3	0	.82	−0.04 (−0.06 to −0.01)	.003	(−0.20 to 0.13)
Europe	2	41.0	.19	−0.03 (−0.29 to 0.24)	.84	—^c^
Intervention type						
Health applications	8	87.3	<.001	−0.14 (−0.31 to 0.03)	.11	(−0.71 to 0.43)
Website-based platforms	4	1.5	.39	−0.10 (−0.23 to 0.03)	.13	(−0.40 to 0.20)
WeChat-based platforms	4	97.2	<.001	−0.60 (−1.10 to −0.10)	.02	(−3.03 to 1.83)
2-hour postprandial blood glucose						
Publication year						
≤2020	4	51.5	.10	0.11 (−0.22 to 0.44)	.50	(−1.15 to 1.38)
>2020	6	91.7	<.001	−0.89 (−1.32,−0.47)	<.001	(−2.40 to 0.61)
Publication country						
Asia	7	90.7	<.001	−0.79 (−1.19 to −0.40)	<.001	(−2.17 to 0.58)
North America	1	0	<.001	0.70 (0.13 to 1.27)	.02	—
Europe	2	0	.37	−0.01 (−0.28 to 0.26)	.94	—
Intervention type						
Health applications	5	94.0	<.001	−0.64 (−1.48 to 0.21)	.14	(−3.89 to 2.62)
Website-based platforms	2	0	.37	−0.01 (−0.28 to 0.26)	.94	—
WeChat-based platforms	3	88.9	<.001	−0.60 (−1.03 to −0.16)	.007	(−5.96 to 4.76)
Gestational weight gain						
Publication year						
≤2020	3	96.8	<.001	−1.00 (−2.36 to 0.36)	.15	(−17.76 to 15.76)
>2020	7	76.5	<.001	−0.68 (−1.45 to 0.09)	.08	(−3.07 to 1.72)
Publication country						
Asia	7	90.3	<.001	−0.87 (−1.62 to −0.12)	.02	(−3.28 to 1.54)
Europe	3	69.9	.04	−0.56 (−1.62 to 0.50)	.30	(−12.44 to 11.33)
Intervention type						
Health applications	9	90.5	<.001	−0.59 (−1.22 to 0.04)	.07	(−2.62 to 1.44)
WeChat-based platforms	1	0	<.001	−2.48 (−3.54 to −1.42)	<.001	—

a MD: mean difference.

b PI: predictive interval.

c Not applicable.

### Publication Bias

Publication bias was not assessed for HbA_1c_, emergency cesarean section, and neonatal respiratory distress because fewer than 10 studies were included for each outcome. For the remaining 8 outcomes, no significant publication bias was detected based on funnel-plot inspection and the Egger regression test. Corresponding funnel plots and Egger regression plots are provided in Multimedia Appendix 4.

## Discussion

### Principal Findings

This meta-analysis synthesized data from 23 RCTs including 3055 pregnant women with GDM to evaluate the effects of telemedicine on maternal and neonatal outcomes. Pooled analyses indicated that telemedicine was associated with reductions in fasting blood glucose levels, 2-hour postprandial blood glucose levels, and gestational weight gain, as well as lower rates of cesarean section, emergency cesarean section, macrosomia, neonatal hypoglycemia, and neonatal respiratory distress. Of note, the pooled estimates for fasting blood glucose, 2-hour postprandial blood glucose, and gestational weight gain were derived from evidence with very high unexplained heterogeneity and PIs crossing the null value. Therefore, these findings should be regarded as suggestive rather than definitive. No statistically significant differences were observed for HbA_1c_, preterm birth, or NICU admission.

### Multidimensional Mechanisms of Telemedicine in the Management of GDM

The positive effects of telemedicine on patients with GDM manifest in multiple aspects. These potential mechanistic explanations may underpin the observed benefits of telemedicine, although they cannot be directly confirmed from the present meta-analysis alone.

First, with wearable devices and mobile apps, telemedicine enables real-time, continuous monitoring of physiological indicators, including blood glucose, in pregnant women and round-the-clock tracking of glycemic data [22,39,40,57]. Once abnormal blood glucose fluctuations are detected, clinical staff can deliver prompt interventions, substantially shortening the duration and mitigating the severity of intrauterine fetal exposure to hyperglycemia, reducing excessive fetal insulin secretion triggered by maternal hyperglycemia, and thereby lowering the risks of neonatal hypoglycemia and fetal overgrowth [44].

Second, telemedicine provides personalized diet and exercise programs according to individual maternal characteristics [42,43,47,48,50,51,55]. Controlling meal order and total caloric intake, promoting moderate exercise, and limiting sedentary time may reduce the risks of macrosomia, neonatal hypoglycemia, and neonatal respiratory distress.

Third, the telemedicine system can automatically integrate multidimensional health data to form continuous, retrievable quantitative records [50,57]. The system enables earlier and more accurate detection of abnormal data and sends out early warnings. Clinical practitioners may adjust intervention protocols in a timely manner, thus realizing refined antenatal management.

Fourth, by leveraging instant messaging, online consultation, and video-based follow-up, telemedicine removes temporal and geographic constraints [45]. When blood glucose levels exceed the recommended range or when unhealthy lifestyle behaviors are identified, the system promptly provides an analysis of underlying causes and corrective guidance, thereby reducing the frequency of in-person hospital visits, alleviating the economic burden on pregnant women, and improving the continuity and compliance of disease management.

Fifth, when combined with behavioral intervention strategies, including cognitive behavioral therapy and health check-in supervision, telemedicine can markedly improve pregnant women’s long-term compliance and self-efficacy regarding blood glucose monitoring, dietary restriction, and regular physical activity. It facilitates the formation of proactive self-management behaviors and reduces the risk of abnormal fetal growth and development [43,46].

Finally, certain telemedicine systems support a self-management model that requires minimal regular manual input from clinicians. This enables pregnant women to intuitively understand the intrinsic correlations between diet, physical activity, and blood glucose levels and independently adjust their lifestyle regimens [43,46,47]. Through sustained long-term control of blood glucose levels and body weight, the intrauterine developmental environment can be optimized, lowering the risks of fetal developmental anomalies and various adverse neonatal complications.

### Comparison With Previous Work

This systematic review showed that telemedicine interventions were associated with improvements in fasting blood glucose and 2-hour postprandial blood glucose levels in patients with GDM but had no statistically significant effect on HbA_1c_ level. However, given the very high and unexplained heterogeneity and the PIs crossing the null for both fasting and 2-hour postprandial glucose levels, these associations should be interpreted as suggestive rather than conclusive. This conclusion is broadly consistent with those of Bertini et al [28], Xie et al [18], El Seifi et al [58], and Yang et al [21]. All of these authors reported that telemedicine interventions significantly improved fasting blood glucose or 2-hour postprandial blood glucose levels. Regarding HbA_1c_, the present study differs from the positive conclusions reported by Xie et al [18], Bertini et al [28], and Eberle et al [59]. Our findings are consistent with those of Rasekaba et al [27] and El Seifi et al [58]. Ming et al found no significant overall difference in HbA_1c_ [19]. Only a mild improvement was observed in their GDM subgroup. Possible reasons for these discrepancies are that HbA_1c_ reflects average blood glucose over 2 to 3 months, whereas the intervention window for GDM is relatively short. The timing of HbA_1c_ measurement and variations in statistical power also differed across studies. In addition, this review found that telemedicine can improve self-management ability in pregnant women with GDM, thereby effectively controlling excessive gestational weight gain. This finding aligns with the results reported by Bertini et al [28].

This systematic review reveals that telemedicine significantly reduces the overall and emergency cesarean delivery rates among women with GDM. This finding is consistent with those reported by El Seifi et al [58], Xie et al [18], and Yang et al [21]. Telemedicine enables the precise regulation of blood glucose levels and gestational weight gain through timely feedback. It decreases the incidence of macrosomia and reduces indications for cesarean delivery, such as dystocia and fetal distress, thereby lowering the risks of elective and emergency cesarean deliveries.

Overall, previous studies have drawn inconsistent conclusions regarding the effects of telemedicine interventions on adverse pregnancy outcomes and neonatal complications [18,19,21,27,58]. Early systematic reviews mostly found no significant benefits. More recent meta-analyses incorporating a greater number of RCTs and larger sample sizes tend to suggest that telemedicine interventions can reduce the risk of gestational hypertension or preeclampsia, premature rupture of membranes, preterm birth, macrosomia, neonatal hypoglycemia, and composite neonatal complications. However, outcomes such as NICU admission rates, neonatal jaundice, and neonatal respiratory distress syndrome mostly showed no significant differences.

### Limitations and Strengths

#### Strengths

This meta-analysis has several strengths. First, it provides an up-to-date synthesis, with searches extending to August 2026 and 23 RCTs involving 3055 women with GDM, capturing recent advances in app-based and multiplatform telemedicine. Second, it evaluates a broad set of clinically actionable perinatal outcomes, including emergency cesarean section, neonatal respiratory distress, and admission to the NICU. These outcomes have been inconsistently reported in prior reviews. Third, this review followed rigorous methodology. It adhered to PRISMA guidelines, was registered in PROSPERO, and used dual independent screening and data extraction. Leave-one-out sensitivity analyses, meta-regression, and subgroup analyses were also used to explicitly explore potential sources of heterogeneity. Fourth, beyond efficacy assessment, this review synthesizes active intervention components and provides actionable implementation considerations for embedding telemedicine within routine antenatal care, especially in resource-limited settings.

#### Limitations

Nevertheless, the present meta-analysis has several limitations. First, studies spanned from 2007 to 2026. Older telemedicine hardware and protocols differ from modern app–based management approaches, which may increase between-study heterogeneity. Importantly, despite sensitivity, subgroup, and meta-regression analyses, very high heterogeneity (*I*²>90%) for fasting glucose, 2-hour postprandial glucose, and gestational weight gain could not be convincingly explained by publication year, publication region, or intervention modality. The corresponding 95% PIs all crossed the null value, indicating that the true effect in future comparable clinical settings might be null or might favor usual care. Therefore, these pooled estimates should be viewed as hypothesis-generating rather than definitive. The clinical benefits of telemedicine for these 3 outcomes should be interpreted cautiously. Second, telemedicine interventions were categorized into 3 types (health applications, website-based platforms, and WeChat-based platforms) and varied in duration. These modalities differ in architecture, interactivity, and feedback. Pooling heterogeneous interventions may obscure best-performing modalities. Although we conducted modality-stratified subgroup analyses, small subgroup sample sizes and mixed model interventions precluded robust comparative conclusions; this remains an important limitation. Third, most studies reported only short-term gestational and immediate neonatal outcomes; thus, long-term offspring benefits could not be assessed. Fourth, we restricted searches to English-language publications and excluded eligible non-English studies, which introduces potential language bias that may compromise completeness of the evidence base and generalizability of findings. Trials reporting favorable effects are more often published in English, whereas null effect studies tend to appear in non–English-language journals. This may inflate pooled estimates for fasting glucose, 2-hour postprandial glucose, and gestational weight gain, consistent with their wide PIs crossing the null value. Systematic differences in intervention delivery across linguistic groups create uncertainty for non–English-speaking settings. Combined with the very low to low certainty of evidence across all outcomes according to GRADE, this bias warrants cautious interpretation of effect sizes. The exclusion of gray literature further carries publication-bias risk.

#### Implications for Clinical Practice

Findings from this meta-analysis suggest that telemedicine interventions may confer clinical benefits for selected maternal and neonatal outcomes among women with GDM. Benefits were observed for cesarean section–related outcomes, macrosomia, and certain neonatal end points, although improvements in glycemic control and gestational weight gain should be interpreted cautiously, given the very high unexplained heterogeneity and crossing 95% PIs. Telemedicine may mitigate limitations of conventional prenatal care such as delayed follow-up and insufficient individualized management. It may be particularly valuable for primary health care settings with limited medical resources. Clinical staff may enhance pregnant women’s self-management adherence via online monitoring and personalized health education. This approach may also potentially lower maternal- and infant-related health care expenditures, though economic outcomes were not formally assessed in this review. The present findings may inform clinical guideline revisions and support the consideration of integrating telemedicine into routine GDM antenatal care. Future research should develop intelligent, tailored intervention programs and implement long-term follow-up to assess offspring health. This work will further refine digital-health approaches for perinatal care.

### Conclusion

This systematic review and meta-analysis included 23 RCTs to investigate the effects of telemedicine interventions on maternal and neonatal outcomes among women with GDM. Pooled analyses suggested that telemedicine may improve fasting glucose levels, 2-hour postprandial blood glucose levels, and gestational weight gain. It may also reduce the incidence of cesarean section, emergency cesarean section, macrosomia, neonatal hypoglycemia, and neonatal respiratory distress. No statistically significant differences were observed for preterm birth or admission to the NICU. Importantly, fasting glucose levels, 2-hour postprandial blood glucose levels, and gestational weight gain showed very high unexplained heterogeneity after sensitivity, subgroup, and meta-regression analyses. Their 95% PIs all crossed the null value. Therefore, these specific findings should be interpreted with particular caution and regarded as preliminary. These results support the potential value of telemedicine for GDM management. Nevertheless, GRADE assessment rated the certainty of evidence as very low to low across all 11 outcomes. Risks of bias, inconsistency, and imprecision reduce confidence in the observed benefits. These limitations warrant caution when drawing firm conclusions regarding the clinical value of telemedicine for GDM. Despite these shortcomings, telemedicine represents a promising intervention approach. It can overcome temporal and geographical barriers, enable real-time monitoring and timely feedback, and deliver personalized dietary and exercise guidance to improve self-management adherence. Telemedicine may serve as a potential supplementary option for routine prenatal care. It may be of particular relevance in resource-limited settings. Future large-sample, long-term follow-up RCTs are needed to further verify long-term maternal and neonatal benefits and identify the optimal intervention modality.

## Supplementary material

10.2196/103569 Multimedia Appendix 1 Deviations from the original protocol in the final review.

10.2196/103569 Multimedia Appendix 2 Search strategy.

10.2196/103569 Multimedia Appendix 3 Grading of Recommendations Assessment, Development, and Evaluation evidence quality assessment.

10.2196/103569 Multimedia Appendix 4 Supplementary forest plot, funnel plot, Egger test, and subgroup analysis.

10.2196/103569 Checklist 1 PRISMA 2020 checklist.

## Abbreviations

GDM: gestational diabetes mellitus
GRADE: Grading of Recommendations Assessment, Development, and Evaluation
HbA_1c_: hemoglobin A_1c_
NICU: neonatal intensive care unit
PI: prediction interval
PICOS: Population, Interventions, Comparison, Outcomes, and Study type
PRISMA: Preferred Reporting Items for Systematic Reviews and Meta-Analyses
PROSPERO: International Prospective Register of Systematic Reviews
RCT: randomized controlled trial
RR: risk ratio
