# Supplementary material for: The Impact of Telemedicine Interventions on Maternal and Fetal Outcomes in Women With Gestational Diabetes: Systematic Review and Meta-Analysis of Randomized Controlled Trials
Source: J Med Internet Res. 2026 Oct 23;28:e103569. doi: 10.2196/103569 (PMC13693963; doi:10.2196/103569)
Supplement: Supplementary file 1 [file jmir-v28-e103569-s001.docx]

**Multimedia Appendix 2. Deviations from the original protocol in the final review**

|  | **Original registration** | **Deviation** | **Explanation** |
| --- | --- | --- | --- |
| **Data-synthesis plan** | “No formal data synthesis is planned ‑ data will be described but not combined.”Planned effect metrics: standardized mean difference (SMD) for continuous outcomes, odds ratio (OR) for dichotomous outcomes. | Formal quantitative meta-analysis was performed using Stata 18.0. For continuous outcomes, mean difference (MD) was selected; for dichotomous perinatal outcomes, relative risk (RR) was applied. Heterogeneity was assessed via Cochran’s Q test and I^2^; random-/fixed-effects models were chosen accordingly. Sensitivity analysis, meta-regression and subgroup analyses were undertaken to explore sources of heterogeneity. | We initially anticipated high heterogeneity and planned only narrative synthesis. After full-text screening, sufficient comparable RCTs with consistent outcome reporting allowed quantitative pooling. MD preserves original clinical units for glycaemic/weight data; RR is preferred over OR for non-rare perinatal events. Additional analyses were applied to explore observed between-study heterogeneity. |
| **Search language restrictions** | There are no language restrictions. | Eligibility restricted to English-language publications only. | Due to limited translation resources, we applied an English-only filter. The risks of resulting language bias are thoroughly discussed within the Limitations section of the main manuscript. |
| **Main sources to be searched** | Embase, MEDLINE, PubMed and Scopus. | Embase, PubMed, Scopus; additionally Cochrane Library, Web of Science were added. Separate MEDLINE search was dropped. | MEDLINE-indexed citations were fully retrieved via the PubMed interface, so no independent MEDLINE search was run. Cochrane Library and Web of Science were supplemented to broaden the literature coverage for this topic. |
| **Main outcomes list** | cesarean section, emergency cesarean section, fasting blood glucose, 2-hour postprandial blood glucose, gestational weight gain, preterm birth, macrosomia, neonatal hypoglycemia, neonatal respiratory distress, admission to the NICU. Hemoglobin A1c (HbA1c) was not listed among outcomes. | Added HbA1c as one prespecified main outcome. | After expanding the search databases, 7 additional eligible RCTs were identified, many of which reported HbA1c. Given the increased available data, HbA1c was incorporated as a clinically relevant glycaemic outcome. |
| **Review team** | Hongyue He, Xiaohong Xu, Lixin Liu. | Expanded author list: Hongyue He, Juan Deng, Jingxian Liu, Xiaohong Xu, Jiao Chen, Lixin Liu, Haoyue Zhang, Fang Wang. | Added co-authors who contributed to literature screening, data extraction, statistical analysis and manuscript writing, consistent with the final manuscript author list. |
| **Review stage** | The review was marked as “planned or ongoing”, with synthesis not yet commenced. | All review stages (searching, screening, extraction, risk-of-bias assessment, data synthesis) marked as completed. | Updated PROSPERO record to reflect the finished status of the systematic review. |
