# Supplementary material for: The Impact of Telemedicine Interventions on Maternal and Fetal Outcomes in Women With Gestational Diabetes: Systematic Review and Meta-Analysis of Randomized Controlled Trials
Source: J Med Internet Res. 2026 Oct 23;28:e103569. doi: 10.2196/103569 (PMC13693963; doi:10.2196/103569)
Supplement: Supplementary file 2 [file jmir-v28-e103569-s002.docx]

**Appendix 1: Search Strategy**

**Search results for each source**

| **Database** | **No. refs found** | **Date searched** |
| --- | --- | --- |
| PubMed | 446 | 15/8/2026 |
| Embase | 993 | 15/8/2026 |
| Cochrane Library | 520 | 15/8/2026 |
| Web of Science | 239 | 15/8/2026 |
| Scopus | 579 | 16/8/2026 |

**Search Strategy for each source**

| Database |  | Search term | Results |
| --- | --- | --- | --- |
| PubMed | #1 | "Telemedicine"[Mesh] OR "Digital Technology"[Mesh] |  |
|  | #2 | "Mobile Health"[Title/Abstract] OR "mHealth"[Title/Abstract] OR "Telehealth"[Title/Abstract] OR "eHealth"[Title/Abstract] OR "Telecare"[Title/Abstract] OR "Telemedicine"[Title/Abstract] OR "Teleconsultation*"[Title/Abstract] OR "Remote Consultation"[Title/Abstract] OR "APP"[Title/Abstract] OR "Mobile App"[Title/Abstract] OR "Application*"[Title/Abstract] OR "Phone*"[Title/Abstract] OR "Smartphone*"[Title/Abstract] OR "Mobile Phone*"[Title/Abstract] OR "Mobile Telephone*"[Title/Abstract] OR "Mobile Devic*"[Title/Abstract] OR "Smartwatch"[Title/Abstract] OR "Email*"[Title/Abstract] OR "Texting*"[Title/Abstract] OR "Text Messag*"[Title/Abstract] OR "Computer*"[Title/Abstract] OR "Website*"[Title/Abstract] OR "Web-based"[Title/Abstract] OR "Software"[Title/Abstract] |  |
|  | #3 | #1 OR #2 | 2984618 |
|  | #4 | "Diabetes, Gestational"[Mesh] |  |
|  | #5 | "Diabetes Mellitus, Gestational"[Title/Abstract] OR "Gestational Diabetes Mellitus"[Title/Abstract] OR"Diabetes, Pregnancy-Induced"[Title/Abstract] OR "Diabetes, Pregnancy Induced"[Title/Abstract] OR "Pregnancy-Induced Diabetes"[Title/Abstract] OR "Gestational Diabetes"[Title/Abstract] |  |
|  | #6 | #4 OR #5 | 33482 |
|  | #7 | "Randomized Controlled Trials as Topic"[Mesh] OR "Controlled Clinical Trials as Topic"[Mesh] OR "Randomized Controlled Trial" [Publication Type] OR "Controlled Clinical Trial"[Publication Type] |  |
|  | #8 | "random*"[Title/Abstract] OR "RCT"[Title/Abstract] |  |
|  | #9 | #7 OR #8 | 2095150 |
|  | #10 | #3 AND #6 AND #9 | 446 |
| Embase | #1 | 'telehealth'/exp OR 'digital technology'/exp |  |
|  | #2 | 'e-health':ti,ab,kw OR 'ehealth':ti,ab,kw OR 'telehealth':ti,ab,kw OR 'telecare':ti,ab,kw OR 'e-care':ti,ab,kw OR 'e-healthcare':ti,ab,kw OR 'virtual care':ti,ab,kw OR 'virtual healthcare':ti,ab,kw OR 'telemedicine':ti,ab,kw OR 'telenursing':ti,ab,kw OR 'e-nursing':ti,ab,kw OR 'virtual nursing':ti,ab,kw OR 'remote consultation':ti,ab,kw OR 'phone*':ti,ab,kw OR 'application*':ti,ab,kw OR 'smartwatch':ti,ab,kw OR 'email*':ti,ab,kw OR 'texting*':ti,ab,kw OR 'computer*':ti,ab,kw OR 'website*':ti,ab,kw OR 'web-based':ti,ab,kw OR 'software':ti,ab,kw |  |
|  | #3 | #1 OR #2 | 3674522 |
|  | #4 | 'maternal diabetes mellitus'/exp |  |
|  | #5 | 'diabetes during pregnancy':ti,ab,kw OR 'diabetes in pregnancy':ti,ab,kw OR 'diabetes mellitus during pregnancy':ti,ab,kw OR 'diabetes mellitus in pregnancy':ti,ab,kw OR 'diabetic mother':ti,ab,kw OR 'maternal diabetes':ti,ab,kw OR 'maternal diabetes mellitus':ti,ab,kw OR 'gestational diabetes':ti,ab,kw OR 'diabetes mellitus gravidarum':ti,ab,kw OR 'diabetes mellitus of pregnancy':ti,ab,kw OR 'diabetes of pregnancy':ti,ab,kw OR 'gestational diabetes mellitus':ti,ab,kw OR 'maternal gestational diabetes mellitus':ti,ab,kw OR 'pregnancy diabetes':ti,ab,kw OR 'pregnancy diabetes mellitus':ti,ab,kw OR 'pregnancy in diabetics':ti,ab,kw OR 'pregnanc* in diabetic*':ti,ab,kw |  |
|  | #6 | #4 OR #5 | 80579 |
|  | #7 | 'randomized controlled trial (topic)'/exp |  |
|  | #8 | 'randomized controlled trial*':ti,ab,kw OR 'pragmatic clinical trials as topic':ti,ab,kw OR 'randomized controlled trials as topic':ti,ab,kw OR 'random*':ti,ab,kw OR 'rct':ti,ab,kw |  |
|  | #9 | #7 OR #8 | 2874551 |
|  | #10 | #3 AND #6 AND #9 | 993 |
| Cochrane Library | #1 | MeSH descriptor: [Telemedicine] explode all trees |  |
|  | #2 | ('telehealth' OR 'ehealth' OR 'telecare' OR 'telemedicine' OR 'telenursing' OR 'mHealth' OR 'APP' OR 'phone' OR 'smartwatch' OR 'digital technology' OR 'Email' OR 'Texting' OR 'Website' OR 'Web-based'):ti,ab,kw |  |
|  | #3 | #1 OR #2 | 71084 |
|  | #4 | MeSH descriptor: [Diabetes, Gestational] explode all trees |  |
|  | #5 | ('maternal diabetes mellitus' OR 'diabetes during pregnancy' OR 'diabetes in pregnancy' OR 'diabetes mellitus during pregnancy' OR 'diabetes mellitus in pregnancy' OR 'diabetic mother' OR 'maternal diabetes' OR 'gestational diabetes' OR 'diabetes mellitus gravidarum' OR 'diabetes mellitus of pregnancy' 'diabetes of pregnancy' OR 'gestational diabetes mellitus' OR 'maternal gestational diabetes mellitus' OR 'pregnancy diabetes' OR 'pregnancy diabetes mellitus' OR 'pregnancy in diabetics' OR 'pregnanc* in diabetic*'):ti,ab,kw |  |
|  | #6 | #4 OR #5 | 8713 |
|  | #7 | MeSH descriptor: [Randomized Controlled Trial] explode all trees |  |
|  | #8 | ('randomized controlled trial*' OR 'pragmatic clinical trials as topic' OR 'randomized controlled trials as topic' OR 'random*' OR 'RCT'):ti,ab,kw |  |
|  | #9 | #7 OR #8 | 1528601 |
|  | #10 | #3 AND #6 AND #9 | 520 |
| Web of Science | #1 | AB=(telehealth or ehealth or telecare or telemedicine or telenursing or mHealth or APP or phone or smartwatch or 'digital technology' or Email or Texting or Website or Web-based) | 429174 |
|  | #2 | AB=('maternal diabetes mellitus' or 'diabetes during pregnancy' or 'diabetes in pregnancy' or 'diabetes mellitus during pregnancy' or 'diabetes mellitus in pregnancy' or 'diabetic mother' or 'maternal diabetes' or 'gestational diabetes' or 'diabetes mellitus gravidarum' or 'diabetes mellitus of pregnancy' or 'diabetes of pregnancy' or 'gestational diabetes mellitus' or 'maternal gestational diabetes mellitus' or 'pregnancy diabetes' or 'pregnancy diabetes mellitus' or 'pregnancy in diabetics' or 'pregnanc* in diabetic*') | 53736 |
|  | #3 | AB=('randomized controlled trial*' or 'pragmatic clinical trials as topic' or 'randomized controlled trials as topic' or 'random*' or 'RCT' ) | 2585910 |
|  | #4 | #1 AND #2 AND #3 | 239 |
| Scopus | #1 | ( TITLE-ABS-KEY ( telehealth ) OR TITLE-ABS-KEY ( ehealth ) OR TITLE-ABS-KEY ( telecare ) OR TITLE-ABS-KEY ( telemedicine ) OR TITLE-ABS-KEY ( telenursing ) OR TITLE-ABS-KEY ( mHealth ) OR TITLE-ABS-KEY ( APP ) OR TITLE-ABS-KEY ( phone ) OR TITLE-ABS-KEY ( smartwatch ) OR TITLE-ABS-KEY ( 'digital technology' ) OR TITLE-ABS-KEY ( email ) OR TITLE-ABS-KEY ( texting ) OR TITLE-ABS-KEY ( WEBSITE ) OR TITLE-ABS-KEY ( web based ) ) | 1619019 |
|  | #2 | ( TITLE-ABS-KEY ( 'maternal diabetes mellitus' ) OR TITLE-ABS-KEY ( 'diabetes during pregnancy' ) OR TITLE-ABS-KEY ( 'diabetes in pregnancy' ) OR TITLE-ABS-KEY ( 'diabetes mellitus during pregnancy' ) OR TITLE-ABS-KEY ( 'diabetes mellitus in pregnancy' ) OR TITLE-ABS-KEY ( 'diabetic mother' ) OR TITLE-ABS-KEY ( 'maternal diabetes' ) OR TITLE-ABS-KEY ( 'gestational diabetes' ) OR TITLE-ABS-KEY ( 'diabetes mellitus gravidarum' ) OR TITLE-ABS-KEY ( 'diabetes mellitus of pregnancy' ) OR TITLE-ABS-KEY ( 'diabetes of pregnancy' ) OR TITLE-ABS-KEY ( 'gestational diabetes mellitus' ) OR TITLE-ABS-KEY ( 'maternal gestational diabetes mellitus' ) OR TITLE-ABS-KEY ( 'pregnancy diabetes' ) OR TITLE-ABS-KEY ( 'pregnancy diabetes mellitus' ) OR TITLE-ABS-KEY ( 'pregnancy in diabetics' ) OR TITLE-ABS-KEY ( 'pregnanc* in diabetic*' ) ) | 98349 |
|  | #3 | ( TITLE-ABS-KEY ( 'randomized controlled trial*' ) OR TITLE-ABS-KEY ( 'pragmatic clinical trials as topic' ) OR TITLE-ABS-KEY ( 'randomized controlled trials as topic' ) OR TITLE-ABS-KEY ( 'random*' ) OR TITLE-ABS-KEY ( RCT ) ) | 3966818 |
|  | #4 | #1 AND #2 AND #3 | 579 |
