# Supplementary material for: The Impact of Telemedicine Interventions on Maternal and Fetal Outcomes in Women With Gestational Diabetes: Systematic Review and Meta-Analysis of Randomized Controlled Trials
Source: J Med Internet Res. 2026 Oct 23;28:e103569. doi: 10.2196/103569 (PMC13693963; doi:10.2196/103569)
Supplement: Supplementary file 3 [file jmir-v28-e103569-s003.docx]

**Author(s):** Hongyue He, Juan Deng, Jingxian Liu, Xiaohong Xu, Jiao Chen, Lixin Liu, Haoyue Zhang, Fang Wang

**Question:** Telemedicine compared to Usual care for GDM patients

**Setting:** Hospitals and homes

**Bibliography:**

| **Certainty assessment** | | | | | | | **№ of patients** | | **Effect** | | **Certainty** | **Importance** |
| --- | --- | --- | --- | --- | --- | --- | --- | --- | --- | --- | --- | --- |
| **№ of studies** | **Study design** | **Risk of bias** | **Inconsistency** | **Indirectness** | **Imprecision** | **Other considerations** | **telemedicine** | **Usual care** | **Relative (95% CI)** | **Absolute (95% CI)** |  |  |
| **fasting glucose** | | | | | | | | | | | | |
| 16 | randomised trials | serious^a,b^ | very serious^c^ | not serious | serious^d^ | none | 1549 | 1506 | - | MD **0.26 mmol/L lower** (0.42 lower to 0.09 lower) | ⨁◯◯◯ Very low^a,b,c,d^ |  |
| **2-hour postprandial glucose** | | | | | | | | | | | | |
| 10 | randomised trials | serious^a,b^ | very serious^c^ | not serious | serious^d^ | none | 706 | 693 | - | MD **0.5 lower** (0.87 lower to 0.14 lower) | ⨁◯◯◯ Very low^a,b,c,d^ |  |
| **HbA1c** | | | | | | | | | | | | |
| 7 | randomised trials | serious^a,b^ | very serious^c^ | not serious | very serious^e^ | none | 426 | 402 | - | MD **0.29 lower** (0.64 lower to 0.07 higher) | ⨁◯◯◯ Very low^a,b,c,e^ |  |
| **gestational weight gain** | | | | | | | | | | | | |
| 10 | randomised trials | serious^a,b^ | very serious^c^ | not serious | serious^d^ | none | 778 | 757 | - | MD **0.78 lower** (1.4 lower to 0.17 lower) | ⨁◯◯◯ Very low^a,b,c,d^ |  |
| **cesarean section** | | | | | | | | | | | | |
| 16 | randomised trials | serious^a,b^ | serious^f^ | not serious | serious^d^ | none | 1208/1549 (78.0%) | 1161/1506 (77.1%) | **RR 0.84** (0.72 to 1.00) | **123 fewer per 1,000** (from 216 fewer to 3 fewer) | ⨁◯◯◯ Very low^a,b,d,f^ |  |
| **emergency cesarean section** | | | | | | | | | | | | |
| 7 | randomised trials | serious^a,b^ | not serious | not serious | serious^d^ | none | 598/1549 (38.6%) | 579/1506 (38.4%) | **RR 0.60** (0.41 to 0.88) | **154 fewer per 1,000** (from 227 fewer to 46 fewer) | ⨁⨁◯◯ Low^a,b,d^ |  |
| **preterm birth** | | | | | | | | | | | | |
| 12 | randomised trials | serious^a,b^ | not serious | not serious | very serious^e^ | none | 999/1549 (64.5%) | 973/1506 (64.6%) | **RR 0.76** (0.57 to 1.01) | **155 fewer per 1,000** (from 278 fewer to 6 more) | ⨁◯◯◯ Very low^a,b,e^ |  |
| **macrosomia** | | | | | | | | | | | | |
| 12 | randomised trials | serious^a,b^ | not serious | not serious | serious^d^ | none | 1158/1549 (74.8%) | 1051/1506 (69.8%) | **RR 0.56** (0.42 to 0.74) | **307 fewer per 1,000** (from 405 fewer to 181 fewer) | ⨁⨁◯◯ Low^a,b,d^ |  |
| **neonatal hypoglycemia** | | | | | | | | | | | | |
| 10 | randomised trials | serious^a,b^ | not serious | not serious | serious^d^ | none | 779/1549 (50.3%) | 755/1506 (50.1%) | **RR 0.77** (0.61 to 0.98) | **115 fewer per 1,000** (from 196 fewer to 10 fewer) | ⨁⨁◯◯ Low^a,b,d^ |  |
| **neonatal respiratory distress** | | | | | | | | | | | | |
| 7 | randomised trials | serious^a,b^ | not serious | not serious | serious^d^ | none | 484/1549 (31.2%) | 475/1506 (31.5%) | **RR 0.65** (0.46 to 0.92) | **110 fewer per 1,000** (from 170 fewer to 25 fewer) | ⨁⨁◯◯ Low^a,b,d^ |  |
| **admission to the NICU** | | | | | | | | | | | | |
| 11 | randomised trials | serious^a,b^ | not serious | not serious | very serious^e^ | none | 692/1549 (44.7%) | 670/1506 (44.5%) | **RR 0.83** (0.62 to 1.11) | **76 fewer per 1,000** (from 169 fewer to 49 more) | ⨁◯◯◯ Very low^a,b,e^ |  |

**CI:** confidence interval; **MD:** mean difference; **RR:** risk ratio

#### Explanations

a. When conducting telemedicine interventions, it is difficult to blind participants and implementers; therefore, none of the studies conducted adopted blinding, resulting in a high risk of performance bias.

b. Most studies did not adequately describe allocation concealment and outcome assessor blinding.

c. There was significant heterogeneity, and the sources of heterogeneity could not be identified through sensitivity analysis, meta-regression, or subgroup analysis.

d. There were significant differences in sample size among the studies. Excessive differences in sample size can significantly increase the risk of downgrading for imprecision by widening the confidence interval under the random-effects model, and may also create "false precision" when dominated by large-sample studies, leading to an underestimation of imprecision.

e. The 95% confidence interval is relatively wide and crosses the "line of no effect" at the same time.

f. The heterogeneity was moderate, and it decreased after sensitivity analysis.
