# Supplementary material for: The Impact of Telemedicine Interventions on Maternal and Fetal Outcomes in Women With Gestational Diabetes: Systematic Review and Meta-Analysis of Randomized Controlled Trials
Source: J Med Internet Res. 2026 Oct 23;28:e103569. doi: 10.2196/103569 (PMC13693963; doi:10.2196/103569)
Supplement: Supplementary file 4 [file jmir-v28-e103569-s004.docx]

**Forest plot (HbA1c, preterm birth, and admission to the NICU)**

HbA1c

preterm birth

admission to the NICU

**Publication bias (Funnel plot and Egger’s test)**

Fasting blood glucose

2-Hour postprandial blood glucose

Gestational weight gain

Cesarean section

Premature birth

Macrosomia

Neonatal hypoglycemia

Admission to the NICU

**Subgroup analysis**

Fasting blood glucose

2-Hour postprandial blood glucose

Gestational weight gain
